# Health Care Staffing Shortages and Potential National Hospital Bed Shortage

**DOI:** 10.1001/jamanetworkopen.2024.60645

**Published:** 2025-02-19

**Authors:** Richard K. Leuchter, Benjo A. Delarmente, Sitaram Vangala, Yusuke Tsugawa, Catherine A. Sarkisian

**Affiliations:** 1 Division of General Internal Medicine and Health Services Research, Department of Medicine, David Geffen School of Medicine at UCLA, Los Angeles, California; 2 Division of Hospital Medicine, Department of Medicine, Greater Los Angeles VA, Los Angeles, California; 3 Department of Health Policy and Management, Fielding School of Public Health, UCLA, Los Angeles, California; 4 VA Greater Los Angeles Healthcare System Geriatric Research Education and Clinical Center (GRECC), Los Angeles, California

## Abstract

This cross-sectional study assesses several possible US hospital bed occupancy scenarios arising from an aging US population over the next decade, based on varying hospitalization rates and staffed hospital bed supply.

## Introduction

Between August 2020 and April 2024, US hospitals were mandated to report weekly occupancy to the Department of Health and Human Services as part of COVID-19 data tracking efforts, providing unprecedented insight into mean daily census and inpatient bed supply across nearly all hospitals nationwide.^1^ In this report, we repurposed this COVID-19 dashboard to describe several possible US hospital bed occupancy scenarios arising from an aging US population over the next decade, while varying hospitalization rates and staffed hospital bed supply.

## Methods

This cross-sectional study was deemed exempt from review by the UCLA institutional review board and did not require informed consent because it did not use patient data. We adhered to the STROBE reporting guideline.

The aging-adjusted annual number of hospitalizations were calculated by multiplying US Census Bureau population projections^2^ for 2025 to 2035 by an age-adjusted hospitalization rate from the 2019 to 2020 National Inpatient Sample.^3^ These future hospitalizations were used to calculate future hospital census:



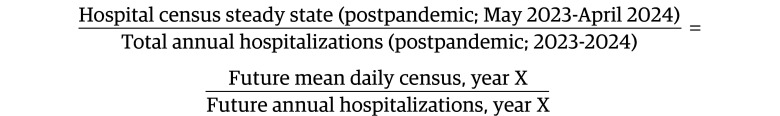



Hospital occupancy for each year between 2025 and 2035 was calculated by dividing mean daily census by staffed hospital bed supply. See eMethods in Supplement 1 for additional methods.

## Results

**Figure 1.**
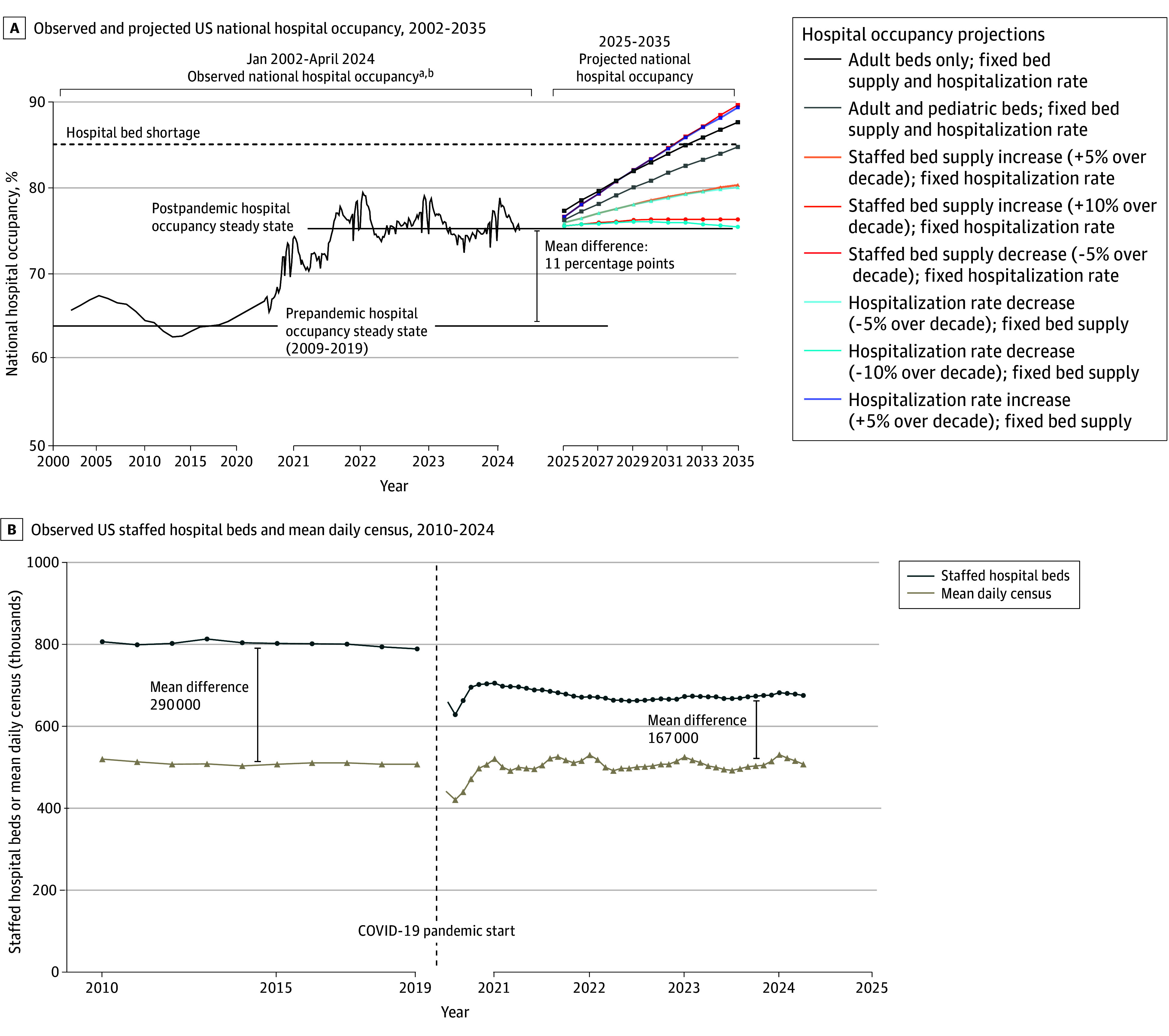
Observed and Projected US National Hospital Occupancy, and Observed US Staffed Hospital Beds and Mean Daily Census A, US hospital occupancy steady state increased from 63.9% pre–COVID-19 pandemic (mean of annual levels 2009-2019) to 75.3% post–COVID-19 public health emergency (mean of weekly levels May 14, 2023, to April 27, 2024). The main series projections account for an increase in the number of hospitalizations from an aging population assuming that neither the staffed hospital bed supply nor the hospitalization rate increase or decrease over the next decade, and demonstrate that a critical hospital occupancy threshold of 85% may be reached by approximately 2032 for adult beds and 2035 for adult and pediatric beds combined. Alternative scenarios modeled aging-adjusted changes in hospitalization rates (−10% to +5% over the next decade) and hospital bed supply (−5% to +10% over the next decade), as described in the legend. B, Hospital occupancy was calculated by dividing mean daily census by the number of staffed hospital beds. During the pre–COVID-19 pandemic steady state (mean of 2009-2019), the number of US staffed hospital beds was 802 000 and the mean daily census was 512 000, as obtained from the American Hospital Association. Both metrics dropped during the early COVID-19 pandemic, and reached respective new post–COVID-19 pandemic steady state (mean of May 2023 to April 2024) of 674 000 and 508 000, according to data reported to the Department of Health and Human Services. The mean difference between staffed hospital beds and mean daily census fell from 290 000 in the prepandemic steady state period to 167 000 in the postpandemic steady state period, which accounts for the increase in hospital occupancy over this time. ^a^Observed staffed hospital beds and mean daily census between 2002 and 2019 were obtained from the American Hospital Association’s Hospital Statistics, 2024 Health Forum, LLC, an affiliate of the American Hospital Association. Mean hospital occupancy for this period was calculated by dividing the average daily census by the number of staffed beds. ^b^Observed mean weekly hospital occupancy, staffed hospital beds, and average daily census between August 2, 2020, and April 27, 2024, were reported by hospitals to the Department of Health and Human Services by mandate and obtained from the Centers for Disease Control and Prevention’s data repository.^1^ Hospital occupancy data from 2020 prior to August 2, and from 2024 after April 27, were not available as these periods were outside of the mandatory hospital occupancy reporting period.

**Figure 2.**
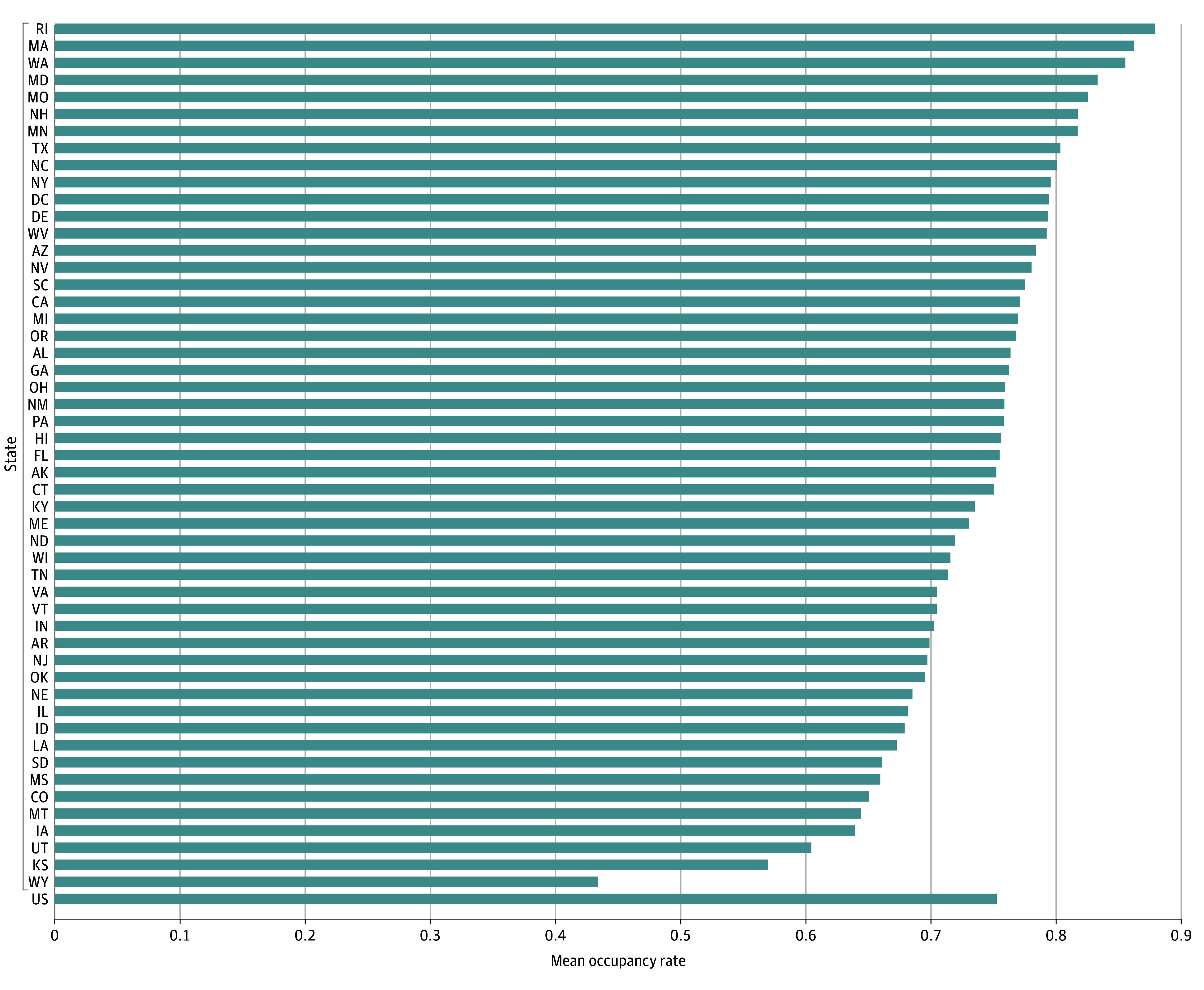
Mean Post–COVID-19 Public Health Emergency Hospital Occupancy by State (May 2023-April 2024) The mean of weekly hospital occupancy in the year following the end of the COVID-19 public health emergency (May 2023 to April 2024) was calculated from data reported to the Department of Health and Human Services.^1^ Horizontal bars represent mean occupancy (mean daily census/staffed hospital beds). There was substantial state-to-state variability in mean postpandemic hospital occupancy, ranging from 43% (Wyoming) to 88% (Rhode Island), with a national mean of 75% (4835 hospitals reporting).

The mean US hospital occupancy was 63.9% (range, 63%-66%) from 2009 to 2019 compared with 75.3% (range, 72%-79%) in the year following the end of the COVID-19 public health emergency (PHE; May 2023 to April 2024) (Figure 1A). The number of staffed hospital beds declined from a prepandemic steady state of 802 000 (2009-2019 mean) to a post-PHE steady state of 674 000, whereas the mean daily census steady state remained at approximately 510 000 (Figure 1B). There was substantial state-to-state variation in the post-PHE hospital occupancy steady state (Figure 2).

Without changes in the hospitalization rate or staffed hospital bed supply, total annual hospitalizations were projected to increase from 36 174 000 in 2025 to 40 177 000 in 2035 with the aging population. This would correspond to a national hospital occupancy of approximately 85% by 2032 for adult beds and by 2035 for adult and pediatric beds combined (Figure 1A).

## Discussion

The US has achieved a new postpandemic hospital occupancy steady state 11 percentage points higher than it was prepandemic. This persistently elevated occupancy appears to be driven by a 16% reduction in the number of staffed US hospital beds rather than by a change in the number of hospitalizations.

Experts in developed countries have posited that a national hospital occupancy of 85% constitutes a hospital bed shortage (a conservative estimate)^4^; our findings show that the US could reach this dangerous threshold as soon as 2032, with some states at much higher risk than others. These scenarios suggest that an increase in the staffed hospital bed supply by 10%, reduction in the hospitalization rate by 10%, or some combination of the two would offset the aging-associated increase in hospitalizations over the next decade.

There are several limitations of these scenarios: (1) they fixed length of stay (LOS) at 2009 to 2019 levels, though this would underestimate future hospital census as LOS has increased since 2019,^5^ (2) they did not account for potential future drastic changes in underlying population health (eg, improvements from medical breakthroughs, declines from increasing cardiometabolic disease or cancer burden), and (3) they did not account for the transferability of hospital and/or geographic resources (eg, intensive care unit beds may or may not be convertible into floor beds, nurses may or may not be able to relocate from California to Rhode Island). With the goal of avoiding substantial excess mortality^6^ associated with a national hospital bed shortage, future research should investigate the determinants of recent reductions in the staffed hospital bed supply (eg, tight health care labor markets, hospital closures), and explore frameworks to improve national health system resilience (eg, distributing resources according to geographic demand, innovative models such as next-day clinics to reduce avoidable hospitalizations).

## Notes

Supplement 1. **eMethods.** Supplemental Methods
eReferences

Supplement 2.
Data Sharing Supplement
