# Supplementary material for: Health Care Staffing Shortages and Potential National Hospital Bed Shortage
Source: JAMA Netw Open. 2025 Feb 19;8(2):e2460645. doi: 10.1001/jamanetworkopen.2024.60645 (PMC11840646; doi:10.1001/jamanetworkopen.2024.60645)
Supplement: Supplement 1. — eMethods. Supplemental Methods eReferences [file jamanetwopen-e2460645-s001.pdf]

Supplemental Online Content

Leuchter RK, Delarmente BA, Vangala S, Tsugawa Y, Sarkisian CA. Health care staffing shortages and potential national hospital bed shortage, 2025-2035. *JAMA Netw Open.* 2025;8(2):e2460645. doi:10.1001/jamanetworkopen.2024.60645

eMethods. Supplemental Methods

eReferences

This supplemental material has been provided by the authors to give readers additional information about their work.

© 2025 Leuchter RK et al. *JAMA Network Open*.

eMethods. SUPPLEMENTAL METHODS

Study Population and Data Sources

Weekly U.S. hospital occupancy (the numbers of total staffed hospital beds and total occupied hospital beds) for adult and pediatric beds from 88-98% of U.S. hospitals were published by the CDC between August 2, 2020-April 27, 2024.[1] During this period, U.S. hospitals were mandated to report occupancy first directly to the U.S. Department of Health and Human Services (HHS) during August 2, 2020-December 14, 2022, and then later to the HHS through the CDC's National Healthcare Safety Network from December 15, 2022-April 27, 2024. Starting May 1, 2024, U.S. hospitals were no longer required to report hospital occupancy and supply data to HHS. Occupancy measurements excluded psychiatric, rehabilitation, religious non-medical, Veterans Administration, Defense Health Agency, and Indian Health Service hospitals. Inpatient hospital beds were defined as those in the facility that at the time of reporting were set-up, staffed, and able to be used for a patient. This analysis excluded critical care (i.e., ICU) beds. Additional details regarding mandatory reporting are available through the HHS.[2]

Historical U.S. hospital occupancy metrics from 2000-2019 were obtained from the American Hospital Association's Hospital Statistics.[3] While the data source changed between 2019-2020, the AHA national hospital occupancy estimates from 2014-2019 uptrended from 62.7% to 64.4%, and the HHS data resumed in August 2020 at a very similar absolute value of 66.8% and continued a similar trajectory over the next 5 months to 71.2% in December 2020. Additionally, the mean average daily censuses obtained by the AHA and HHS during their respective reporting periods were nearly identical: 512,000 v. 508,000. These points suggest that despite possible methodological differences in reporting staffed

© 2025 Leuchter RK et al. *JAMA Network Open*.

hospital beds and hospital census, the relative value of these may be preserved and comparable between AHA and HHS data.

National hospitalization rates per 100,000 people stratified by age band (0-17, 18-44, 45-64, 65-74, 75+) are publicly available from the National Inpatient Sample (NIS),[4] a 20% sample of all-payer non-critical care discharges from all hospitals that participate in the Healthcare Cost and Utilization Project (HCUP). We used the two most recent years of hospitalization rates available through HCUP Fast Stats (2019-2020), which yielded a conservative estimate of contemporary hospitalization rates: 2019 is the most recent year of NIS data not impacted by the pandemic (and thus most likely reflective of immediately post-pandemic hospitalization rates), and 2020 hospitalization rates were artificially lowered by the Covid-19 pandemic. Furthermore, the use of 2019 hospitalization rates is supported by the fact that the 2024 average daily census returned to 2019 levels (Figure 1b), suggesting that hospitalization rates were similar between these two years. Mean 2019-2020 hospitalization rates by age band per 100,000 people were: 0-17, 6,690; 18-44, 6,924; 45-64, 9,586; 65-74, 17,328; 75+, 32,587.

U.S. population estimates for 2023-2034 were obtained from the official U.S. Census Bureau population projections, which account for trends in fertility, mortality, and net migration.[5] The Main Series estimates were used for the scenarios in this study.

Statistical Analysis

Hospitalization Projections

To estimate number of hospitalizations for 2025-2035, we multiplied the annual U.S. Census Bureau's population estimate for five age bands (0-17, 18-44, 45-64, 65-74, 75+) by the corresponding NIS 2019-2020 hospitalization rate for that age band. We explored the possibility of both decreases and increases in the 2019 hospitalization rate within the alternative scenario projections.

Occupancy Projections: Main Series

The main series hospital occupancy projections (Figure 1a) were calculated using the formula detailed in the main manuscript text. These projections were completed for all adult and pediatric beds combined, as well as just adult beds. The main series projections assessed the effects of an aging U.S. population on hospital occupancy, given that older adults exhibit hospitalization rates nearly five times higher than younger adults.[4] These projections assumed that the staffed hospital bed supply was fixed at the observed levels in the year after the Covid-19 Public Health Emergency (PHE; May 14, 2023-April 27, 2024), and that hospitalization rates were fixed at 2019-2020 levels. Alternative scenarios for changes in both staffed hospital bed supply and hospitalization rates are detailed below.

Occupancy Projections: Alternative Scenarios

<u>Alternative hospitalization rates</u>: We evaluated how hospital occupancy may change if the 2019-2020 hospitalization rate were to increase/decrease by 5% or 10% between 2025-2035. These scenarios would be representative of changes in underlying population health (either increases or decreases), sudden shocks that increased hospitalization rates up to 5%, or the introduction of new technologies and/or health system innovations to reduce hospitalizations by up to 10%. We repeated the same method in *Hospitalization Projections* above but increased/decreased the 2019 hospitalization rate by 0.5% or 1.0% per year for each year between 2025-2035. This produced modified hospitalization estimates, which were used to produce hospital occupancy projections using the same methods used in the main series occupancy projections. These projections assumed that the hospital bed supply was fixed at 2023-2024 levels over this period, and did take an aging U.S. population into account.

<u>Alternative staffed hospital bed supply:</u> We evaluated how hospital occupancy may change if the number of staffed hospital beds were to contract/expand by 5% or 10% between 2025-2035. These scenarios would be representative of changes in labor supply (e.g., more or fewer nurses) or changes in

© 2025 Leuchter RK et al. *JAMA Network Open*.

bed supply (e.g., complete hospital closures or temporary ward closures). We used the mean observed total bed supply from May 14, 2023-April 27, 2024 published by the CDC[1] as a baseline (674,214 beds) and then increased/decreased the baseline number of beds by 0.5% or 1.0% per year for each year from 2025-2035. We divided the aging-adjusted projected hospital census from the main series by this number to obtain projected hospital occupancy. These projections also accounted for the aging U.S. population's effect on hospital census.

All alternative scenarios were calculated for all non-critical care hospital beds (adult and pediatric beds combined), rather than adult beds alone.

Hospital Bed Shortage Assumptions

Consistent with CMS and other researchers we used an 85% occupancy threshold to defined a bed shortage,[6–8] a conservative estimate given that adverse events arising from hospital overcrowding start to occur well below an 85% threshold (especially among smaller and rural hospitals).[9,10] Previously published studies in developed countries estimate that overcrowded hospitals or understaffed hospital wards is associated with a *relative* increase in in-hospital mortality of 2-5% per hospitalization,[11–14] which is a reasonable estimate given that overnight stays in the ED can increase relative in-hospital mortality by nearly 40% in older adults.[15] Thus, the Discussion also assumes that a hospital bed shortage is associated with excess mortality.

Projection Assumptions

We made two key assumptions common to all occupancy projections. The first was that the mean hospital occupancy in the year following the end of the Covid-19 PHE (May 15, 2023- April 27, 2024) is the new post-pandemic hospital occupancy steady state. It was reasonable to benchmark all projections against this average occupancy for multiple reasons. First, this period is four full years out from the start of the Covid-19 pandemic, so it is unlikely to be a pandemic-related anomaly. Second, the

© 2025 Leuchter RK et al. *JAMA Network Open*.

percentage of hospitalizations for Covid-19 never exceeded 5% during this period, with a mean percentage of hospitalizations for Covid-19 of only 1.9%.[1] Third, a mean national hospital occupancy around 75% has been sustained since July 2021,[1] again making this level more likely to be a persistent trend rather than a transient anomaly.

The second key assumption was that hospital length of stay (LOS) remained stable over the projection period. This was a reasonable assumption given that national LOS has remained unchanged at 6.1-6.2 days between 2009-2019,[16] and has actually increased since 2019.[3,17]

The assumptions specific to each projection are discussed above with the methods relevant to that given projection. We also acknowledge that these projections of a national hospital occupancy are subject to ecologic fallacy whereby trends in national hospital utilization may not hold true on state, county, or hospital levels. To better understand this potential source of bias, state-level occupancies are depicted in Figure 2.

© 2025 Leuchter RK et al. *JAMA Network Open*.

eREFERENCES

1. Centers for Disease Control and Prevention (CDC). Weekly United States Hospitalization Metrics by Jurisdiction, During Mandatory Reporting Period from August 1, 2020 to April 30, 2024, and for Data Reported Voluntarily Beginning May 1, 2024, National Healthcare Safety Network (NHSN). Published 2024. Accessed June 11, 2024. https://data.cdc.gov/Public-Health-Surveillance/Weekly-United-States-Hospitalization-Metrics-by-Ju/aemt-mg7g/about_data

2. U.S. Department of Health and Human Services. *Guidance for Hospitals and Acute Care Facilities Reporting of Respiratory Pathogen, Bed Capacity, and Supply Data to CDC's National Healthcare Safety Network (NHSN)*.; 2023. Accessed June 9, 2024. https://www.cdc.gov/nhsn/covid19/hospital-reporting.html.

3. AHA Hospital Statistics™ | AHA Data. Accessed October 1, 2024. https://www.ahadata.com/aha-hospital-statistics

4. Agency for Healthcare Research and Quality (AHRQ), Healthcare Cost and Utilization Project (HCUP). Fast Stats Data Tools – National Hospital Utilization & Costs. Accessed January 11, 2023. https://datatools.ahrq.gov/hcup-fast-stats/

5. U.S. Census Bureau. 2023 National Population Projections Datasets. Accessed November 1, 2023. https://www.census.gov/data/datasets/2023/demo/popproj/2023-popproj.html

6. Green L V. How Many Hospital Beds? *Inq J Heal Care Organ Provision, Financ*. 2002;39(4):400-412. doi:10.5034/inquiryjrnl_39.4.400

7. Janke AT, Melnick ER, Venkatesh AK. Hospital Occupancy and Emergency Department Boarding During the COVID-19 Pandemic. *JAMA Netw Open*. 2022;5(9):e2233964-e2233964. doi:10.1001/JAMANETWORKOPEN.2022.33964

8. Kelen GD, Wolfe R, D'onofrio G, et al. Emergency Department Crowding: The Canary in the Health Care System. *NEJM Catal*. Published online 2021. doi:10.1056/CAT.21.0217

9. Pratt AC, Wood RM. Addressing overestimation and insensitivity in the 85% target for average bed occupancy. *Int J Qual Heal Care*. 2021;33(3):mzab100. doi:10.1093/intqhc/mzab100

10. Proudlove NC. The 85% bed occupancy fallacy: The use, misuse and insights of queuing theory. *Heal Serv Manag Res*. 2020;33(3):110-121. doi:10.1177/0951484819870936

11. Sharma N, Moffa G, Schwendimann R, Endrich O, Ausserhofer D, Simon M. The effect of time-varying capacity utilization on 14-day in-hospital mortality: a retrospective longitudinal study in Swiss general hospitals. *BMC Health Serv Res*. 2022;22(1):1-11. doi:10.1186/S12913-022-08950-Y/TABLES/2

12. Hsuan C, Segel JE, Hsia RY, Wang Y, Rogowski J. Association of emergency department crowding with inpatient outcomes. *Health Serv Res*. 2023;58(4):828-843. doi:10.1111/1475-6773.14076

13. Madsen F, Ladelund S, Linneberg A. High levels of bed occupancy associated with increased inpatient and thirty-day hospital mortality in Denmark. *Health Aff*. 2014;33(7):1236-1244. doi:10.1377/HLTHAFF.2013.1303/ASSET/IMAGES/LARGE/2013.1303FIGEX3.JPEG

14. Rubbo B, Saville C, Dall'Ora C, et al. Staffing levels and hospital mortality in England: a national

© 2025 Leuchter RK et al. *JAMA Network Open*.

panel study using routinely collected data. *BMJ Open*. 2023;13(5):e066702. doi:10.1136/BMJOPEN-2022-066702

15. Roussel M, Teissandier D, Yordanov Y, et al. Overnight Stay in the Emergency Department and Mortality in Older Patients. *JAMA Intern Med*. 2023;183(12):1378-1385. doi:10.1001/JAMAINTERNMED.2023.5961

16. National Center for Health Statistics. *Hospital Admission, Average Length of Stay, Outpatient Visits, and Outpatient Surgery, by Type of Ownership and Size of Hospital: United States, Selected Years 1980–2019*.; 2020.

17. The Issue Issue Brief: Patients and Providers Faced with Increasing Delays in Timely Discharges. Accessed October 7, 2024. www.aha.org

18. Abir M, Goldstick J, Malsberger R, Bauhoff S, Setodji CM, Wenger N. The Association Between Hospital Occupancy and Mortality Among Medicare Patients. *Jt Comm J Qual Patient Saf*. 2020;46(9):506-515. doi:10.1016/J.JCJQ.2020.05.003

19. Centers for Disease Control and Prevention (CDC). CDC COVID Data Tracker: Hospital Capacity. Published 2023. Accessed June 3, 2024. https://covid.cdc.gov/covid-data-tracker/#hospital-capacity

20. French G, Hulse M, Nguyen D, et al. Impact of Hospital Strain on Excess Deaths During the COVID-19 Pandemic — United States, July 2020–July 2021. *MMWR Morb Mortal Wkly Rep*. 2021;70(46):1613-1616. doi:10.15585/MMWR.MM7046A5

21. Cummings P. The Relative Merits of Risk Ratios and Odds Ratios. *Arch Pediatr Adolesc Med*. 2009;163(5):438-445. doi:10.1001/ARCHPEDIATRICS.2009.31

© 2025 Leuchter RK et al. *JAMA Network Open*.
