# Supplementary material for: Health Care Staffing Shortages and Potential National Hospital Bed Shortage
Source: JAMA Netw Open. 2025 Feb 19;8(2):e2460645. doi: 10.1001/jamanetworkopen.2024.60645 (PMC11840646; doi:10.1001/jamanetworkopen.2024.60645)
Supplement: Supplement 2. — Data Sharing Supplement [file jamanetwopen-e2460645-s002.pdf]

Data Sharing Statement

Leuchter. Health Care Staffing Shortages and Potential National Hospital Bed Shortage. *JAMA Netw Open*. Published February 19, 2025. doi:10.1001/jamanetworkopen.2024.60645

Data
Data available: Yes
Data types: Other (please specify)
Additional Information: All data is publicly available
How to access data: All data is publicly available
When available: With publication

Supporting Documents
Document types: None

Additional Information
Who can access the data: All data is publicly available
Types of analyses: All data is publicly available
Mechanisms of data availability: All data is publicly available
Any additional restrictions: All data is publicly available
